# Scientific integrity among nursing students participating in the Scientific Initiation Program: An exploratory study

**DOI:** 10.1590/S1980-220X2018047703548

**Published:** 2020-04-06

**Authors:** Natállia Rodrigues Araújo da Silva, Gabriela Cristina Cantisani Pádua, Maria Rita Carvalho Garbi Novaes, Dirce Bellezi Guilhem

**Affiliations:** 1 Universidade de Brasília, Faculdade de Ciências da Saúde, Programa de Pós-Graduação em Ciências da Saúde, Brasília, DF, Brazil.; 2 Fundação de Ensino e Pesquisa em Ciências da Saúde, Escola Superior de Ciências da Saúde, Brasília, DF, Brazil.; 3 Universidade de Brasília, Faculdade de Ciências da Saúde, Departamento de Enfermagem, Brasília, DF, Brazil.

**Keywords:** Scientific Misconduct, Ethics, Research, Ethical Review, Morals, Students, Nursing, Má Conduta Científica, Ética em Pesquisa, Revisão Ética, Princípios Morais, Estudantes de Enfermagem, Mala Conducta Científica, Ética en Investigación, Revisión Ética, Principios Morales, Estudiantes de Enfermería

## Abstract

**Objective:**

To know the positions and practices adopted by nursing students in scientific initiation programs about the principles of scientific integrity in the different stages of the process of doing science.

**Method:**

An exploratory study of a quantitative nature, in which nursing student participants of the Scientific Initiation Program from the Federal District were interviewed.

**Results:**

Fifty (50) nursing students participated in the study. Most of the interviewed participants presented good notions about the process of conducting research in its different stages. Nevertheless, it was found that even though they were familiar with good scientific practices, students did not always behave in the most responsible manner. It was observed that the knowledge on topics related to the ethics of the scientific process was predominantly obtained through formal education, consisting of classes and courses. Nonetheless, the importance of complementary spaces such as research and research groups is recognized.

**Conclusion:**

Research experiences are important educational and vocational training spaces for students. Therefore, good research practices need to be included early in the academic curriculum.

## Resumo

**Objetivo:**

Conhecer posicionamentos e práticas adotados por alunos de enfermagem inseridos em programas de iniciação científica acerca dos princípios de integridade científica nas diferentes etapas do processo de fazer ciência.

**Método:**

Estudo exploratório, de natureza quantitativa, em que foram entrevistados estudantes de enfermagem do Distrito Federal, participantes do Programa de Iniciação Científica.

**Resultados:**

Participaram do estudo 50 estudantes de enfermagem. A maioria dos entrevistados apresentou boas noções sobre o processo de condução de pesquisa, em suas diferentes etapas. Apesar disso, verificou-se que, mesmo possuindo familiaridade com boas práticas científicas, nem sempre os estudantes se comportavam de maneira mais responsável. Observou-se que os conhecimentos sobre temas relacionados à eticidade do processo científico foram obtidos predominantemente por meio de ensino formal, constituído por aulas e disciplinas. Apesar disso, reconhece-se a importância de espaços complementares, como a pesquisa e grupos de pesquisa.

**Conclusão:**

As experiências em pesquisa são importantes espaços educativos e de formação profissional para os estudantes. Por isso, boas práticas em pesquisa precisam ser incluídas precocemente no currículo acadêmico.

## Resumen

Conocer planteamientos y prácticas adoptados por alumnos de enfermería incluidos en programas de iniciación científica acerca de los principios de integridad científica en las distintas etapas del proceso de hacer ciencia.

**Método:**

Estudio exploratorio, de naturaleza cuantitativa, en que fueron entrevistados estudiantes de enfermería del Distrito Federal, participantes en el Programa de Iniciación Científica.

**Resultados:**

Participaron en el estudio 50 estudiantes de enfermería. La mayoría de los entrevistados presentaron buenas nociones acerca del proceso de conducción de la investigación, en sus distintas etapas. Pese a ello, se verificó que, aun teniendo familiaridad con buenas prácticas científicas, no siempre los estudiantes se portaban de modo más responsable. Se observó que los conocimientos acerca de los temas relacionados con la etnicidad del proceso científico fueron obtenidos predominantemente mediante enseñanza formal, constituida de clases y asignaturas. Sin embargo, se reconoce la importancia de espacios complementarios, como la investigación y los grupos de investigación.

**Conclusión:**

Las experiencias en investigación son importantes espacios educativos y de formación profesional para los estudiantes. Por lo que las buenas prácticas en investigación necesitan incluirse precozmente en el currículo académico.

## INTRODUCTION

Nursing is a health profession that has care as its epistemological object of know-how. The care process embraces three basic dimensions: care, education-research, and administrative-managerial. Each has its own body of knowledge and strategies for its development and implementation. The second aspect of education-research assumes fundamental importance in training new professionals and to promote knowledge production which subsidizes care^(1)^.

Scientific production in the nursing field in recent years has significantly contributed to its recognition as a workforce and source of theoretical and practical knowledge. This was due to an educational evolution through strengthening research groups and qualifying researchers^(2)^.

The development of research and the search for establishing a unique body of knowledge are effective strategies for strengthening nursing as a science and profession^(1,2)^. Thus, it is essential to enable human resources in undergraduate and graduate studies to qualify the profession through both critical and investigative thinking^(2–3)^.

With the numerical growth of productions and the inclusion of young researchers in the research scenario, the need to discuss issues related to scientific integrity and research ethics arises. As science is a human activity, it is subject to researchers’ own interests and dishonest practices^(4)^. Although scientific knowledge has correction mechanisms through peer verification and review (for example), errors arising from scientific misconduct can lead to social and economic losses, poor quality of publications and scientific losses^(4–5)^.

Ethical discussion is a legitimate concern in teaching future nursing professionals and researchers. The dissemination of these contents should be focused on building values, attitudes and skills which are essential for professional practice and scientific practice, beyond conceptual debates^(6)^.

The discussion about scientific integrity becomes fundamental for responsible scientific exercise. This concept encompasses principles related to honesty, responsibility, ethics, impartiality, transparency, objectivity, truthfulness and reliability, which should be applied at all stages of the investigative process^(4,7)^.

The issue of scientific integrity started to gain discussion in the 1980s. Important fraud scandals involving US researchers were revealed in that decade, which raised alert from research institutions and society to the problem of scientific misconduct^(8)^.

The definition of scientific integrity revolves around two conceptions: one moral and one normative. Morality is based on the idea of probity, honesty and righteousness, which rest on a positive interpretation of values. In the normative conception, scientific integrity is treated as a responsibility, an inherent duty to scientific exercise as a way to guarantee the quality and transparency of science^(9)^.

Strategies for training and forming ethics in researchers have been widely discussed by research funding agencies. As an example, the US National Institute of Health (NIH) has developed the Responsible Conduct of Research (RCR), a training tool for students and researchers to receive instruction in the research ethics process in both formal and informal settings^(10)^.

Initiatives such as the Scientific Initiation Program (*Programa de Iniciação Científica* – *PROIC*) of Brazilian the National Council for Scientific and Technological Development (*Conselho Nacional de Desenvolvimento Científico e Tecnológico* – *CNPq*) contribute to inserting undergraduate students in the universe of scientific production. This helps in capacitating and training human resources for research and to improve future professionals^(2–3,11)^.

Thus, scientific initiation provides new opportunities for students, such as the development of critical skills which are inherent to the research process, and contributes to continuous learning, always having the prerogative of ethical responsibility in conducting research and professional practice^(12)^.

Being aware of the knowledge and behaviors adopted by young scientists in the process of preparing and conducting research and disseminating knowledge through scientific publications helps in identifying failures in their conduct and in implementing ethical guidelines regarding scientific production.

This study aimed to know the positions and practices adopted by nursing students inserted in scientific initiation programs about the principles of scientific integrity in the different stages of the process of doing science: study design and conception, ethical review of the protocol, research conduct and dissemination of results.

## METHOD

### Study design

This is an exploratory-descriptive quantitative study implementing a cross-sectional design with a non-probabilistic-intentional sample.

### Sample

Nursing undergraduate students from the Federal District were interviewed, including students from public and private institutions.

Participants of the Scientific Initiation Program for the three years of 2013–2014, 2014–2015 and 2015–2016 were included.

### Data collection

Data were obtained by applying a research instrument adapted for the study. The original instrument was aimed at evaluating the opinion and perceptions of Brazilian nursing and health researchers about scientific integrity^(4)^, and was built and adapted from a previous study^(13)^ developed on the theme and through a survey in the literature. The original instrument was submitted to an evaluation process by 4 researchers (two international and two national) specializing in the thematic area.

Data collection was performed by completing a structured questionnaire composed of 27 objective questions. The instrument was divided into four sections: I) general data of the participants; II) questions to classify the level of agreement or disagreement regarding the statements about the production, conduct and publication of research process; III) self-report items about the participant’s behavior as a researcher/undergraduate research fellow; and IV) items on ethical training and scientific integrity.

The first part of the questionnaire contained questions related to gender, age, educational institution, semester in the course, number of participantions in scientific initiation, participation modality in the program and fluency in a second language.

The second section of the questionnaire contained a total of 12 questions relating to the process of preparing, conducting and publishing research results. Respondents should demonstrate their level of agreement or disagreement with the assertions on a scale from 1 to 5 according to the Likert scale, ranging from “strongly disagree” to “strongly agree”.

The third part of the research instrument contained a total of 4 questions about behaviors adopted by students as undergraduate researchers. In each question participants could indicate the frequency of a certain attitude, with 3 options: “never occurred”, “occurred once” or “occurred more than once”.

The last section contained a total of 3 questions, in which participants should select the options considered most appropriate for each case.

Data collection was performed in two ways: in person and by access to the specific electronic form of the research.

The in-person collection took place during the 11^th^ Congress of Scientific Initiation of the Federal District, which took place in November 2014 at the Universidade de Brasilia (UnB). Participants were approached and invited to fill out the research instrument and then deposit the instrument in one of the urns located at strategic points in the event location. The urn technique^(14)^ aimed to ensure confidentiality about the origin of respondents’ data and offer greater privacy in completing the questionnaire.

The second procedure for data collection was through access to the electronic form, made available on a page constructed for the study. Participants were informed of the email address of the form and were able to respond to it anonymously. The electronic form was available for completion for three months from January to March 2016, and a response percentage of around 30% was obtained among those who were invited.

The contacts (e-mails) of the potential research participants were obtained through authorization from the Scientific Initiation Program Coordination in each of the educational institutions in which nursing students participated in the program. The invitation was sent up to four times to potential participants. The data were included in the research database after completing the instrument.

Individuals who responded to the electronic form after the data collection period in the case of the electronic procedure, and those who did not report any answer in the questionnaire in person or electronically were excluded.

### Data analysis and processing

Statistical analysis was performed using Excel software, which enabled elaborating descriptive statistics in order to obtain information regarding the profile and perceptions of the participants on the theme of scientific integrity.

### Ethical aspects

The research project was submitted for review and approved by the Research Ethics Committee of the Humanities Institute of the Universidade de Brasilia, under protocol no. 341.345, approved in 2013. It followed the recommendations of the National Health Council Resolution 466/2012. The survey did not cause any harm or risk to the participants, and confidentiality of the information provided by the respondents was guaranteed and there was no possibility of identifying the participants. The data were kept by the lead researcher.

As this research is a delicate subject related to behaviors and ethical conceptions of young researchers, the Research Ethics Committee was asked to waive the Informed Consent Form as a way to guarantee the confidentiality and anonymity of the collected answers.

However, essential information was provided to the research participants in person and digitally. Explanations of the study were made available on the research page for the electronic data collection. The signatures or information that could identify the participants were waived in this procedure.

## RESULTS

After analyzing the questionnaires, the results were grouped into 4 categories: I) demographic and academic characteristics; II) adherence to ethical requirements related to the process of doing science; III) behaviors adopted in developing research; IV) acquisition of knowledge about ethics and scientific integrity.

### Demographic and academic characteristics

In total, 50 undergraduate nursing students participated in the study; 31 (62%) came from the on-site data collection and 19 (38%) through access to the electronic form. Students from five educational institutions answered the survey, being 41 (82%) from public institutions and 9 (18%) from private institutions.

There were 41 (82%) females and 9 (18%) males among the participants. Their average age was 25 years, ranging from 20 to 50 years. Regarding the course semester, 9 (18%) of the respondents were between the 4^th^ and 6^th^ semesters of the course, 35 (70%) were between the 7^th^ and 10^th^, 4 (8%) did not answer the question, and 2 (4%) had already graduated.

Regarding the scientific initiation modality, half of the respondents (n = 25) participated in the program in the voluntary modality and the other half (n = 25) in the paid modality. Among the 50 participants, 36 (72%) had only one participation in the program, and 14 (28%) reported having participated twice or more in the scientific initiation.

Regarding fluency in a second language, 31 (62%) students said they had mastery of a second language and 19 (38%) denied fluency in a second language. English was the reference language for 29 (94%) people among the participants with mastery of another language.

These characteristics are summarized in Table 1.

### Adherence to ethical requirements related to the process of doing science

Table 2 presents the assertions addressed in this part of the instrument.

**Figure 1 –:**
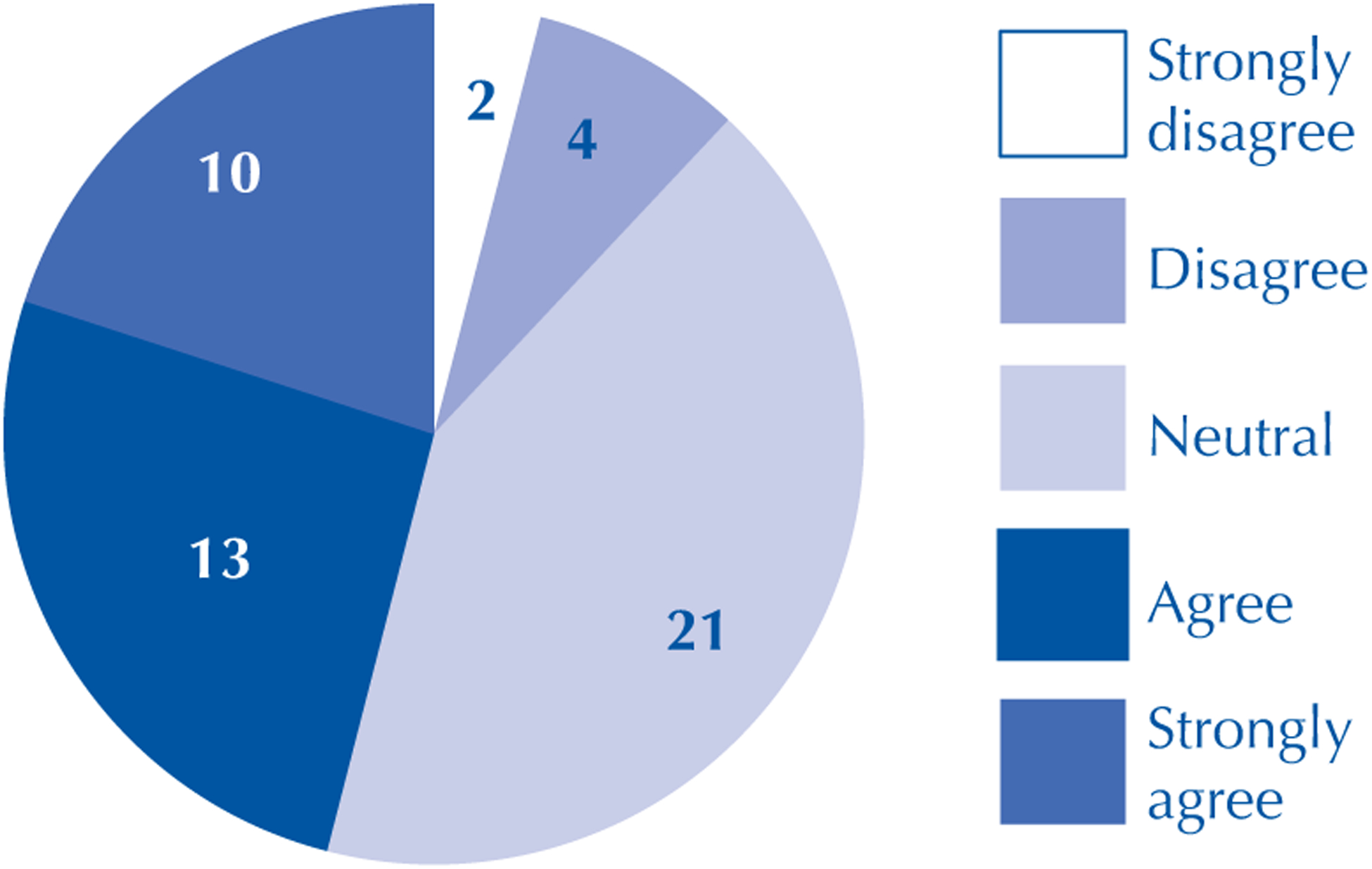
Opinion of participants who would report scientific misconduct – Federal District, Brazil, 2014–2016.

In the seventh question in the section (“Would you report a colleague if you witnessed an act of misconduct?”), 2 (4%) of the respondents strongly disagreed, 4 (8%) disagreed, 21 (42%) were neutral, 13 (26 %) agreed and 10 (20%) strongly agreed (Figure 1).

Although students are well acquainted with good scientific practices regarding the production and research dissemination stages, it was observed that a significant contingent declared neutrality in the hypothetical case situation of reporting a colleague. If we consider the respondents who disagreed and those who were neutral, there would be a total of 27 (54%) students who would not report ethical misconduct by a colleague or would omit it. This data demonstrates that only training or familiarity with recommended behaviors do not guarantee adoption of ethically responsible behaviors.

### Behaviors adopted in research development

Table 3 presents the results of this section.

### Knowledge acquisition on ethics and scientific integrity

In the first question participants were asked to indicate which definitions of plagiarism they considered most appropriate. In total 7 options were made available and students could mark as many as they thought were correct. Among the respondents, 46 (92%) associated plagiarism with the practice of fully copying other authors’ ideas without including due credit, 37 (74%) believe plagiarism is a criminal offense, and 36 (72%) consider it plagiarism when quoting literal passages from other authors without quoting them. Among participants, 35 (70%) associated plagiarism to disrespecting third party copyrights, 26 (52%) believe that plagiarism can be characterized as the practice of reorganizing another author’s ideas while maintaining the general context without including their name, 23 (46%) associated plagiarism with the use of other people’s ideas, and finally 18 (36%) believe that using the same type of construction (argumentation or examples) present in another authors’ text characterizes the practice.

**Figure 2 –:**
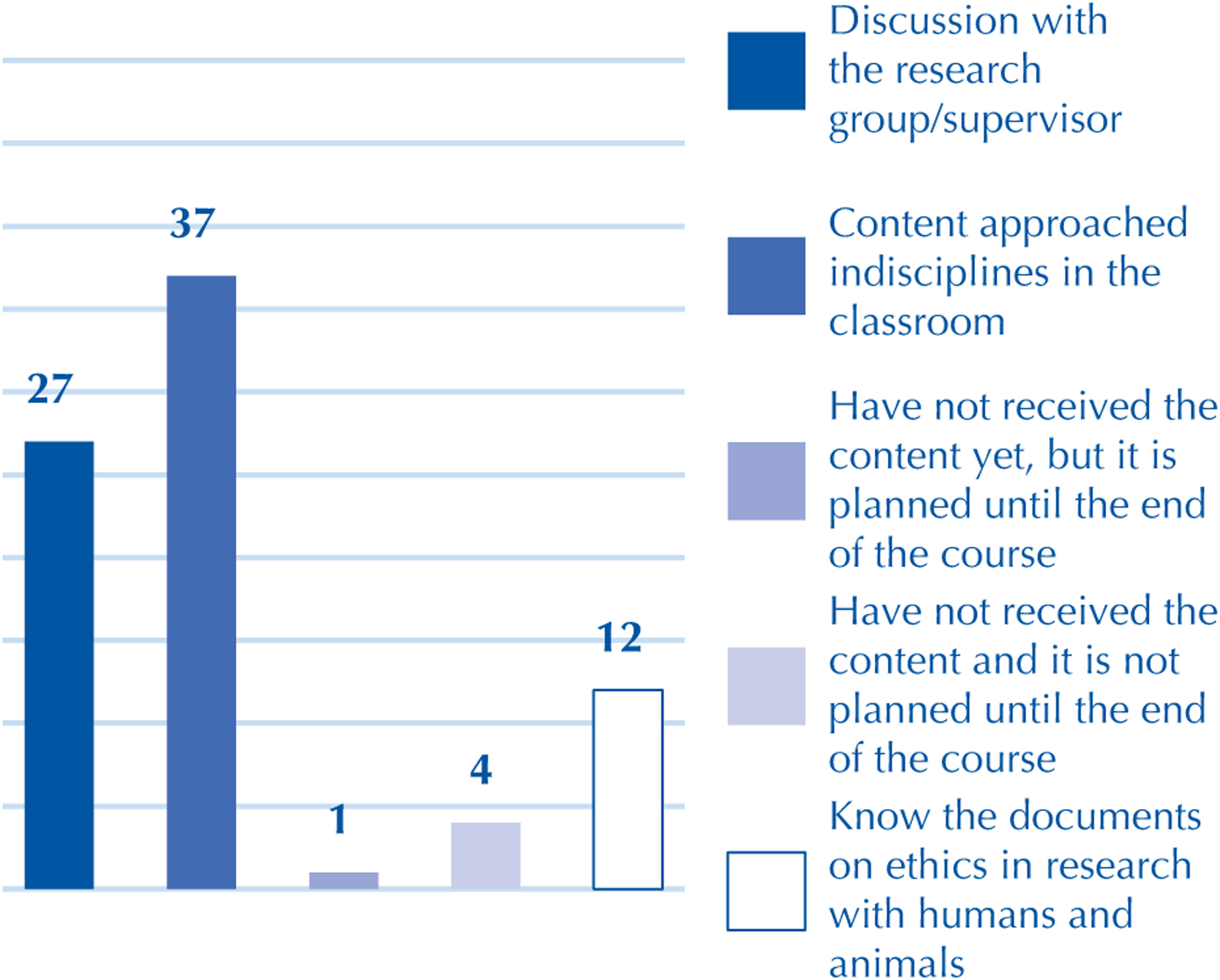
Sources of information on ethics and scientific integrity among undergraduate nursing students – Federal District, Brazil, 2014–2016.

The second question dealt with sources of information on ethics and scientific integrity for students. Discussions on the subject in classroom courses were the main formative sources for 37 (74%) participants, while 27 (54%) of respondents have discussions on the topic with the research group and/or the advisor, 1 (2%) participant said they had not had this content yet but reported that it is planned in a course throughout the curriculum, 4 (8%) said they did not have the content and that it is not planned until the end of the course, and only 12 (24%) participants said they knew the documents on ethics in research with humans and animals (Figure 2).

The results showed a predominance of formal teaching of ethics in courses throughout the curriculum. The content discussed in the classroom is the main source of information on ethics and scientific integrity for 37 (74%) respondents. In addition, the effectiveness of this training should be considered, as only 12 (24%) respondents were familiar with the documents on ethics in research with humans and animals.

It is important to not only question the teaching and knowledge building models, but the quality of these problematizations. Despite these findings, it is necessary to emphasize the role of spaces such as research and research groups to discuss these themes, since moments with a supervisor or research group were important for establishing ethical debates for 27 (54%) participants.

The last question dealt with the disclosure of cases of scientific misconduct reported by the media: 27 (54%) of the participants said they knew about cases of deviation reported by the media and 23 (46%) were unaware of this type of disclosure.

## DISCUSSION

The participants generally presented adequate decision--making regarding the presented situations related to the research practice scenarios. Nevertheless, specific data indicate that there is a tendency to adopt deviant and questionable behaviors in relation to specific situations, such as the report of ethical deviance committed by a colleague.

Colombian research has pointed to a number of factors related to deviant behaviors in research development. With regard to individual behaviors, issues such as irresponsibility and immorality emerged. In those related to interpersonal attitudes, actions were indicated as a “climate of complicity” among peers, who adopt protective and solidarity attitudes in certain conflicting situations during the investigations^(15)^.

An international study conducted with health researchers mainly from the United States, Canada and Europe aimed to measure the frequency of questionable research practices and pointed to alarming situations in the context of scientific practice. Among study participants, 90% reported at least one type of questionable research practice, approximately 18% of researchers had previously ignored the use of unreliable data, and 26.2% had ignored misinterpretation of data by their colleagues^(16)^. Other indicated controversial behaviors were related to improper authorship, citing unread articles, disregarding information provided by participants, plagiarism, and data fabrication. Despite the particularities of the Brazilian research scenario, these data may be correlated with the findings of this research, as the results indicated complacency among researchers when they encountered ethical failures in the research process.

The classic triad of scientific misconduct of fabrication, falsification, and plagiarism had a low prevalence among participants. Nevertheless, with regards to plagiarism, it was observed that most respondents still only associate it with the practice of fully copying ideas without including credits, since limiting ideas on what configures plagiarism remain.

Deviations considered “minor”, such as those related to authorship, publication or reporting of ethical deviations, are situated in the so-called “gray area” of scientific deviations. These behaviors are less serious violations of the ethical precepts of research and often overlooked in the ethical training of researchers^(17–18)^.

The authors argue that while questionable practices have a different impact on data fraud and falsification, which directly affect the quality of scientific findings, these behaviors occur at a much higher frequency among researchers and should therefore also be systematically fought against^(16)^.

Scientific initiation represents a primordial tool for qualifying and training students for their scientific careers, and therefore it is necessary to adopt ethical and scientific requirements for the program qualification. This process involves learning and familiarization with the scientific method, including the ethical care inherent in research work. Student education therefore needs to encompass the dissemination of information on good scientific practice, including recognizing behaviors and deviations that should be avoided in the process of doing science and knowledge production^(3,19)^.

The training of future graduates depends on both individual and social aspects, and university admission represents the beginning of their professional socialization. The moral development of an individual is continuous, the capacity for moral judgment improves according to one’s experience, and the context of each student is the foundation for their professional training^(20–21)^. Undergraduation represents an opportunity to expand the portfolio of experiences of future professionals, being a fertile space for consolidating their moral formation^(20–21)^.

The educational process is dynamic and the student is at the center of it. Thus, the opportunity to outline other formative paths beyond those provided in the formal spaces of the courses represents a significant gain for the student, either through supervised internships or through extension or research projects^(3,6,20)^.

It is essential to provide students with opportunities for reflection and practical application of learned concepts. Ethics and moral judgment are built on practice and interaction. The university, as a locus of training, has a fundamental role in offering experiences which enable students to apply the concepts seen in a dialectical, transversal and critical-reflexive manner. Thus, research, discussion groups and case discussions are great opportunities to do so^(20–21)^.

A research limitation to be highlighted is the reduced sample size in the exploratory study.

## CONCLUSION

Doing science is a process of building autonomy, it is becoming the subject of your learning process. It is essential that knowledge producers are able and equipped with ethical and humanistic guidelines in line with social needs.

Universities and research centers, as human resources trainers, share the task of spreading a culture of ethical and scientific responsibility. Educational institutions are spaces for training and transformation, which in addition to qualifying professionals and scientists in the theoretical scope, also assume the important function of providing practical experiences of knowledge construction, reflection and development of independence.

The results of this study demonstrate the constant need for discussion of ethical issues and scientific integrity at the earliest opportunity in the curriculum. Beyond this movement, it is necessary to rethink the teaching models so that students, as knowledge reproducers, are able to develop capacity to criticize it, revise it and build new ways which fit their reality.

Knowledge production enables changes to occur in the individual and intellectual scope of the subject, but also allows changes in their environment and culture, hence the importance of knowledge as a transformation agent. Solidifying ethical competences with students’ technical--scientific skills in practical experience favors creating a new way of seeing and living their professional identity.

This should be a collective process shared between training centers, teachers, counselors and students. To rethink the inclusion of ethics themes in undergraduate and graduate curricula is to consider the positive impacts on the scientific process and its contributions to the academic and social worlds, aiming at education on good scientific practices in order to curb the deviations which hinder advances in science and society.

## Figures and Tables

**Table 1 –:** Demographic and academic characteristics of undergraduate nursing students from 5 public and private educational institutions of the Federal District, Brazil, 2014–2016.

Characteristics	n (%)
**Gender**	
Male	9 (18)
Female	41 (82)
**Age range (years)**	
20–29	40 (80)
30–39	5 (10)
40–50	3 (6)
No response	2 (4)
**Educational institution**	
Public	41 (82)
Private	9 (18)
**Semester**	
1 ^st^–3^rd^ semesters	0 (0)
4^th^–6^th^ semesters	9 (18)
7^th^–10^th^ semesters	35 (70)
Graduated	2 (4)
Did not respond	4 (8)
**Scholarship holders**	
Paid	25 (50)
Voluntary	25 (50)
**Participation in scientific initiation**	
Once	36 (72)
Twice or more	14 (28)
**Second language**	
Yes	31 (62)
No	19 (38)

**Table 2 –:** Researchers’ opinion on the Process of Preparing, Conducting, and Publishing Research Results – Federal District, Brazil, 2014–2016.

Assertions	SD n (%)	D n (%)	N n (%)	A n (%)	SA n (%)
It is correct to appropriate other people’s writings.	48 (96)	2 (4)	-	-	-
It is correct to take ownership of someone else’s data.	41 (82)	6 (12)	-	-	3 (6)
It is correct to receive credit for someone else’s ideas.	43 (86)	7 (14)	-	-	-
It is correct to be the author of another researcher’s article without collaborating/participating in the work.	44 (88)	4 (8)	1 (2)	1 (2)	-
It is correct to include non-participating authors in an article you produced.	42 (84)	6 (12)	2 (4)	-	-
If it is not possible to collect the research data, it is correct to manufacture or falsify the data to meet the deadlines.	45 (90)	5 (10)	-	-	-
Would you report a colleague if you witnessed an act of misconduct?	2 (4)	4 (8)	21 (42)	13 (26)	10 (20)
Ethical behavior must be present during the conception, proposition and research phases.	1 (2)	-	-	5 (10)	44 (88)
Ethical behavior must be present in the communication phase of the research results.	1 (2)	-	-	6 (12)	43 (86)
Publishing articles is best practice for sharing results.	-	2 (4)	3 (6)	21 (42)	24 (48)
Human research should only be initiated after approval by the Research Ethics Committee.	1 (2)	2 (4)	1 (2)	8 (16)	38 (76)
Animal research must be approved by an Animal Use Committee.	-	2 (4)	2 (4)	4 (8)	42 (84)

A: I agree; SA: I strongly agree; D: disagree; SD: strongly disagree; N: neutral

**Table 3 –:** Self-reported behaviors of undergraduate nursing researchers – Federal District, Brazil, 2014–2016.

Behavior	Never n (%)	Once n (%)	More than once n (%)
Plagiarism	48 (96)	1 (2)	1 (2)
Start data collection prior to approval by the Research Ethics Committee	44 (88)	6 (12)	-
Use third party ideas without crediting them	48 (96)	2 (4)	-
Author or co-author an article without having contributed to its production	45 (90)	5 (10)	-
